# Bismuth selenide nanoparticles enhance radiation sensitivity in colon cancer cells in-vitro

**DOI:** 10.1016/j.bbrep.2024.101732

**Published:** 2024-05-21

**Authors:** Hossein Khosravi, Hamed Manoochehri, Abbas Farmany, Alireza Khoshghadam, Hassan Rafieemehr, Rasool Azmoonfar

**Affiliations:** a Department of Radiology, School of Allied Medical Sciences, Hamadan University of Medical Sciences, Hamadan, Iran; b The Persian Gulf Marine Biotechnology Research Center, The Persian Gulf Biomedical Sciences Research Institute, Bushehr University of Medical Sciences, Bushehr, Iran; c Department of Dental Implant Research, Hamadan University of Medical Sciences, Hamadan, Iran; d Department of Radiooncology, Mahdieh Charity Center, Hamadan University of Medical Sciences, Hamadan, Iran; e Department of Laboratory Sciences, School of Allied Medical Sciences, Hamadan University of Medical Sciences, Hamadan, Iran

**Keywords:** Radiotherapy, Colon cancer, Radiosensitizer, Bismuth selenide, Nanoparticles

## Abstract

**Background:**

Radiotherapy is one of the primary treatments for cancer, but it can cause damage to normal tissues and lead to side effects. The use of radiosensitizers can enhance the sensitivity of cancer cells to radiation, thereby reducing the amount of radiation required and minimizing damage to healthy tissues. Bismuth selenide nanoparticles (Bi2Se3 NPs) have been shown to have potential as radiosensitizers.

**Materials and methods:**

In this study, we investigated the potential of Bi2Se3 NPs as a radiosensitizer in colon cancer cells (HCT-116) in vitro. The cells were treated with various concentrations of Bi2Se3 NPs and then exposed to ionizing radiation. The viability of the cells was assessed using the MTT assay, and the survival rate was evaluated.

**Results:**

Our results showed that Bi2Se3 NPs significantly enhanced the sensitivity of colon cancer cells to ionizing radiation in a dose-dependent manner. The combination of Bi2Se3 NPs and radiation resulted in a significant decrease in cell viability and survival rate compared to radiation alone.

**Conclusion:**

Bi2Se3 NPs have the potential to be used as a radiosensitizer in the treatment of colon cancer. The findings of this study suggest that combining Bi2Se3 NPs with radiation may enhance the effectiveness of radiotherapy and reduce the mortality rate associated with colon cancer. Further studies are needed to investigate the safety and efficacy of this approach in vivo.

## Highlights

• Bismuth selenide nanoparticles (Bi2Se3 NPs) have potential as radiosensitizers in cancer treatment.
• Bi2Se3 NPs significantly enhance the sensitivity of colon cancer cells to ionizing radiation in a dose-dependent manner.
• Combining Bi2Se3 NPs with radiation results in a significant decrease in cell viability and survival rate compared to radiation alone.
• Bi2Se3 NPs have the potential to reduce the mortality rate associated with colon cancer and enhance the effectiveness of radiotherapy.

## 1 Introduction

Rectal cancer, also known as colon cancer, refers to the proliferation of cancerous cells in the colon or rectum, which is a part of the large intestine [1,2]. It is most common form of malignancy and is classified as one of the most lethal types, along with breast, prostate, and lung cancer [3,4]. The recommended treatments for colon cancer consist of a combination of surgery, radiation therapy(RT), chemotherapy, and targeted therapy. Colon cancer ranks as the third most widespread form of cancer [5,6].

RT is a common treatment for cancer that is frequently used alongside surgery and chemotherapy [7]. However, it has the drawback of killing both healthy and cancerous cells when administered in high doses [8]. In RT, it is essential to minimize radiation exposure to healthy tissues and target the tumor cells. Also, sensitizing tumor cells enables higher radiation doses to be delivered to the tumor while minimizing damage to healthy tissues [9,10]. Sensationalizing tumor cells enables higher radiation doses to be delivered to the tumor while minimizing damage to healthy tissues [11]. This balance between radiation protection and sensationalizing tumor cells ensures optimal therapeutic efficacy and patient well-being in RT.

RT faces challenges in treating cancer due to the tumor tissue's resistance to radiation and the need to avoid damaging normal tissue [12,13]. Consequently, enhancing radiosensitivity remains a persistent obstacle in cancer treatment.

With the progress of nanotechnology, nanoparticles have been utilized to enhance the radiation sensitivity of cancer cells [11,14]. Nanoparticles, which are small particles ranging from 1 to 100 nm in size, can easily pass through the cell membrane, with those smaller than 50 nm being particularly effective [15]. This has led to the widespread use of nanoparticles in cancer treatments, where they have been shown to modify the radiation-sensitivity profile of cancer cells and increase the efficacy of radiation therapy [16,17].

Recently, numerous scientists have been concentrating on high atomic number(Z) nanomaterials as radiosensitizers for radiotherapy. This focus is due to the higher stopping power of high-Z metal nanoparticles for ionizing radiation than soft tissue, resulting in enhanced radiotherapy efficacy [11]. The use of Bi2Se3 NPs to enhance radiation sensitivity in colon cancer cells is a promising area of research. Bi2Se3 NPs have been shown to possess properties that make them effective in combination therapy for cancer. Macrophage-membrane-camouflaged hollow Bi2Se3 NPs facilitated photothermal sensitivity and inhibited lung metastasis of breast cancer [18].

Additionally, bismuth oxide nanoparticles have been investigated for their effects on reactive oxygen species (ROS) generation in colon cancer cells, showing promise as radiosensitizers for proton beam therapy [19]. Furthermore, decorated ultrathin bismuth selenide nanosheets have been explored as targeted theranostic agents for in vivo imaging guided cancer radiation therapy [20]. This body of evidence highlights the significance of Bi2Se3 NPs in enhancing the effectiveness of radiation therapy in cancer treatment.

## 2 Materials and methods

### 2.1 Preparation of cell line

The HCT-116 cell line was procured from the Hamedan University of Medical Sciences cell bank and cultured in RPMI-1640 medium supplemented with 10 % FBS. The control group comprised of peripheral blood lymphocytes obtained from a normal individual. Venous blood was collected on heparin anticoagulant, and the mononuclear cells (MNCs) were isolated by centrifugation on ficoll. The MNC-containing supernatant was collected and washed twice with PBS buffer before culturing the lymphocyte cells in RPMI-1640 medium with 10 % FBS, along with K562 cells.

### 2.2 Preparation of Bi2Se3 NPs

In this study, the synthesis of Bi2Se3 NPs was carried out using an aqueous medium. Specifically, a mixture of 1.72 g SeO2, 1.2 g BiCl3, and 0.7 g EDTA was stirred with 135 ml of water, followed by the addition of 1.166 g NaOH and 1.166 g ascorbic acid. The resulting mixture was stirred at 100 rpm and heated in an oil bath at 150 °C for 48 h. Following completion of the reaction, the black precipitate was washed with distilled water and absolute ethanol to remove impurities. The average diameter of these Bi₂Se₃ nanoparticles was approximately 25 nm.

### 2.3 Determining the optimal concentration of nanoparticles

In this study, the synthesis of Bi2Se3 NPs was conducted using an aqueous medium. A mixture of SeO2, BiCl3, and EDTA was stirred with water, followed by the addition of NaOH and ascorbic acid. The resulting mixture was heated and stirred for 48 h, after which the black precipitate was washed with distilled water and ethanol.

To determine the optimal concentration of Bi2Se3 NPs for therapeutic use, different concentrations of the nanoparticles were added to the culture medium of cancer and normal cells. The lethality ratio of the nanoparticles for malignant cells compared to healthy cells was evaluated at different time intervals. The optimal concentration was then used in further tests to assess the effect of the nanoparticles on cancer cells.

### 2.4 Irradiation

For this study, 15 samples of the HCT-116 cell line were utilized and categorized into different groups based on treatment and radiation. The control group (Group 1) received no treatment methods, while Group 2 and Group 3 underwent radiation treatment of 2Gy and 4Gy single fractions, respectively. Group 4 was treated with Bi2Se3 NPs, while Group 5 and Group 6 received a combination of Bi2Se3 NPs and radiation treatment of 2Gy and 4Gy single fractions, respectively. Once the samples reached a confluence level of at least 90 %, the cell culture flasks were irradiated at Mahdieh Radiotherapy Center using a 6 MeV linear accelerator with a single fraction dose of either 2Gy or 4Gy.

### 2.5 MTT assay

In this study, the HCT-116 cell line was utilized and divided into different groups based on treatment and radiation. The control group (Group 1) did not receive any treatment, while Group 2 and Group 3 were subjected to radiation treatment with a single fraction dose of 2Gy and 4Gy, respectively. Group 4 received Bi2Se3 NPs, while Group 5 and Group 6 were given a combination of Bi2Se3 NPs and radiation treatment with a single fraction dose of 2Gy and 4Gy, respectively. When the cell cultures reached a confluence level of at least 90 %, they were irradiated at Mahdieh Radiotherapy Center using a 6 MeV linear accelerator. To assess cell viability, an equal volume of MTT solution was added to the existing media in the culture, followed by incubation at 37 °C for 3 h. After incubation, MTT solvent was added, and the plate was wrapped in foil and shaken on an orbital shaker for 15 min. The absorbance was read at OD = 590 nm after 1 h, and four replicate readings were averaged for each sample.

### 2.6 Apoptotic assay

We employed an Annexin V-FITC/PI Assay kit (Sigma-Aldrich) to identify apoptotic and necrotic cells. Cultured cells were seeded at a density of 2 × 10^5 cells per well in a six-well plate. After 24 and 48 h of treatment, the cells were detached, centrifuged at 300 g for 5 min, and washed twice with PBS. Subsequently, we added a diluted Annexin V binding solution to achieve a final cell concentration of 1 × 10^5 cells/ml. A 100 μl aliquot of the resulting cell suspension was transferred to a new tube. Next, we introduced 5 μl of Annexin-conjugated V-fluorescein isothiocyanate (FITC) and 5 μl of propidium iodide (PI) to the cell suspensions, followed by a 15-min incubation at room temperature in the dark. Finally, we assessed the samples using a flow cytometer. FITC and PI were excited at 488 nm wavelength, with FITC emission detected at 525 nm and PI at 650 nm. Annexin V/PI-negative cells correspond to healthy cells, while Annexin V-positive and PI-negative populations indicate cells in early apoptosis. Annexin V/PI-positive cells represent late apoptosis or secondary necrosis. Therefore, Annexin V-positive cells encompass both early and late apoptosis/secondary necrosis.

### 2.7 Statistical analysis

Descriptive statistics were used to characterize the groups, and statistical analysis was performed using one-way analysis of variance (ANOVA) and paired student t-test in SPSS 20 with a significance level of 0.05. All results were presented as the mean ± Standard deviation (SD).

## 3 Results

### 3.1 The survival rate of cells in the control group and nanoparticles treatment group

The present study investigated the effect of Bi2Se3 NPs in combination with radiation therapy on the viability of HCT-116 colon cancer cells. The control group demonstrated a high survival rate, indicating the aggressive nature of cancer cells without treatment. However, the survival rate of cells in the treatment group with nanoparticles did not differ significantly from the control group, suggesting that Bi2Se3 NPs are not toxic to the culture medium and can be safely combined with radiation therapy to enhance its effectiveness against cancer cells. The small size of the nanoparticles may contribute to their non-lethal effect on cells. These findings suggest that Bi2Se3 NPs hold promise as a potential therapeutic option for colon cancer treatment.

### 3.2 Survival rate of cells after radiation treatment

**Fig. 1:**
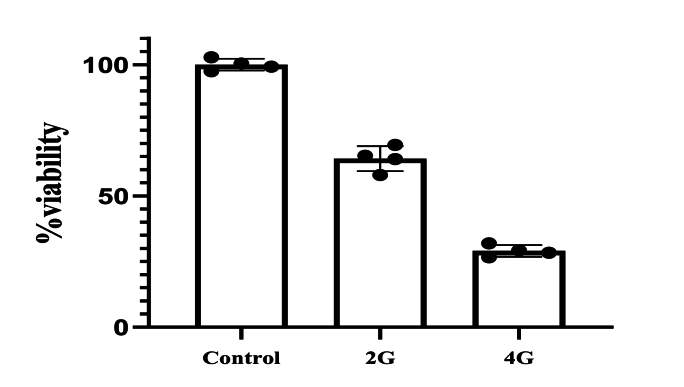
The survival rate of cells after radiation treatment.

**Fig. 2:**
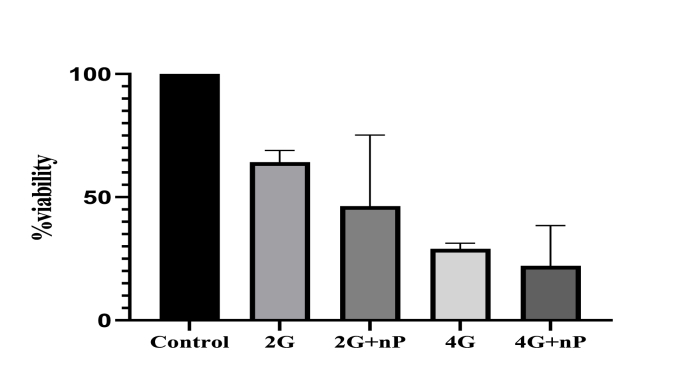
Viability in different groups.

**Fig. 3:**
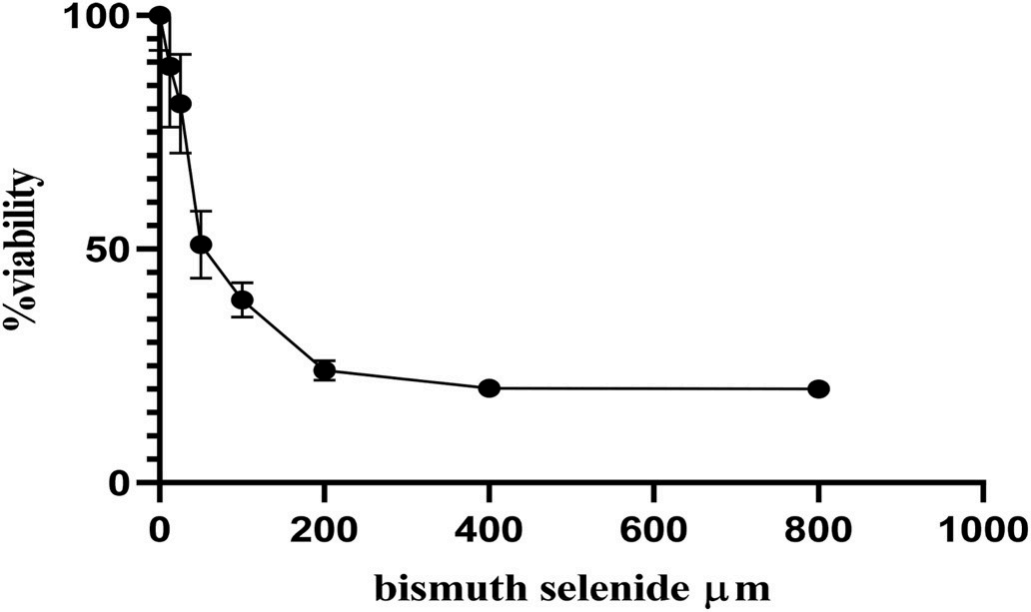
Viability after radiation treatment of 2Gy along with treatment by bismuth selenide nanoparticles.

**Fig. 4:**
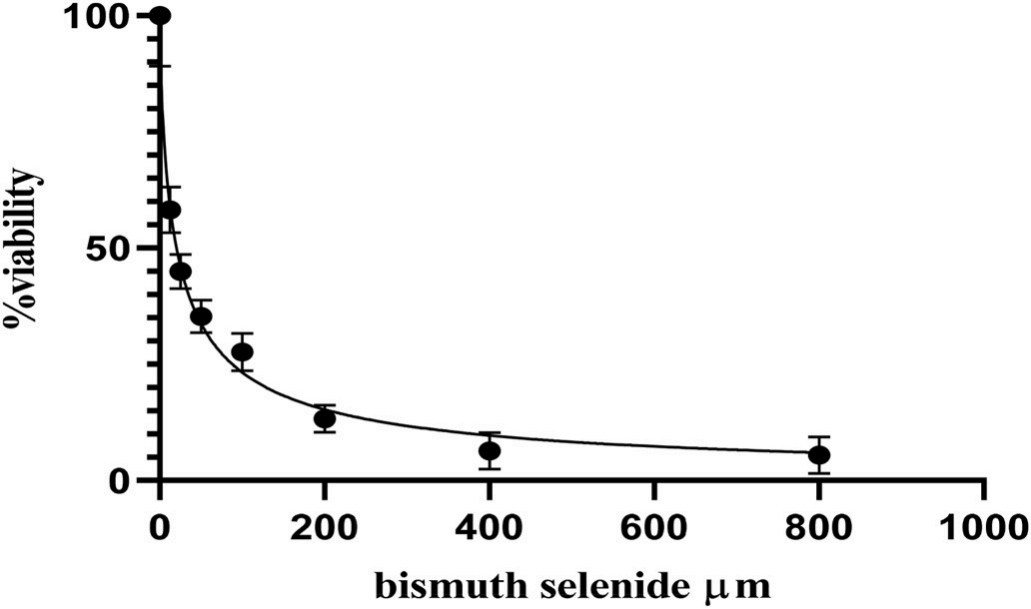
Viability after radiation treatment of 2Gy along with treatment by bismuth selenide nanoparticles.

The survival rate of cultured cells decreased by 36 ± 4 % and 72 ± 2.7 % following radiation treatment with 2Gy and 4Gy single fractions, respectively, compared to the control group that did not receive Bi2Se3 NPs or radiation. Although higher radiation intensity resulted in increased mortality among cancer cells and a significant difference (P < 0.05) was observed between the survival rate of cells in the control group and the radiation treatment groups, it can be inferred that radiation therapy alone is insufficient for complete treatment and efficacy in the highly proliferative colon cancer cell line (Fig. 1) (see Fig. 2) (see Fig. 3) (see Fig. 4).

### 3.3 Effect of Bi2Se3 NPs on viability after radiation treatment

Cell viability, which refers to the number of healthy cells in a sample, and cell proliferation, which is a crucial indicator for understanding the mechanisms involved in cell survival or death after exposure to toxic agents, were assessed. The IC50, which represents the concentration of a compound that causes a 50 % reduction in cell viability or growth, was determined following radiation treatment with 2Gy and 4Gy. Specifically, the IC50 values were found to be 73.15 ± 0.7 and 20.39 ± 0.32, respectively.

Treatment with Bi2Se3 NPs in combination with radiation treatments of 2Gy and 4Gy resulted in a decrease in the survival rate of rectal cancer cells. Moreover, an increase in the dose of nanoparticles in the culture medium led to an enhancement of the effect of radiation treatment. The significant difference (P < 0.05) observed between the survival rates of cells in different groups suggests that radiation treatment increased the mortality of cancer cells and that Bi2Se3 NPs further augmented this effect.

For instance, radiation treatment with a single fraction of 2Gy reduced the survival rate by 64 %, whereas the combination of this treatment with a concentration of 200 μmol of Bi2Se3 NPs in the culture medium led to a 24 % decrease in the survival rate. The presence of these nanoparticles in the culture medium increased the sensitivity of cells to radiation treatment, thereby enhancing its efficacy.

### 3.4 Bi2Se3 nanoparticles enhance X-ray-induced apoptosis in cancer cells

The apoptosis rate of the cells exposed to Bi2Se3 NPs and X-rays was measured by Annexin V-FITC/PI staining and flow cytometry. The results showed that the combination of Bi2Se3 NPs and X-rays significantly increased the percentage of apoptotic cells compared to the control group or the groups treated with either Bi2Se3 NPs or X-rays alone. The apoptotic cells were mainly in the early stage of apoptosis, as indicated by the Annexin V-positive and PI-negative staining. This suggests that Bi2Se3 NPs enhanced the X-ray-induced cell death by triggering the intrinsic apoptotic pathway.

**Fig. 5:**
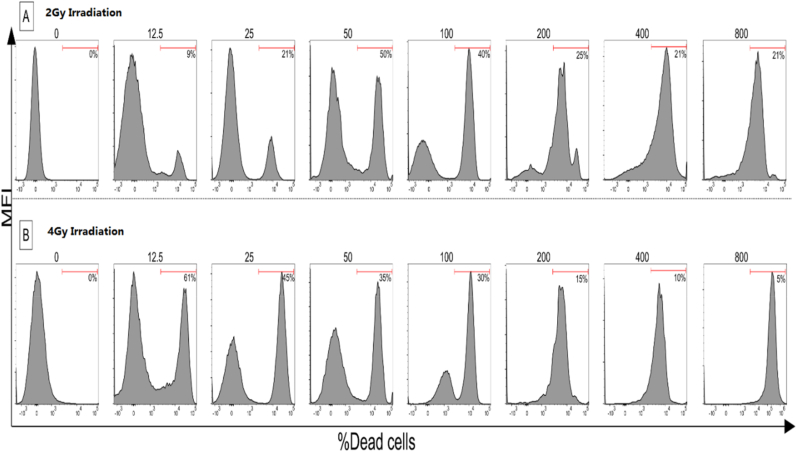
Flowcytometric analysis of cell death using Annexin V-FITC/PI Assay.

The results, reveal a dose-dependent increase in cell death with escalating radiation doses. The histograms demonstrate higher percentages of dead cells in the presence of Bi2Se3 nanoparticles, indicating enhanced radiation sensitivity. These findings contribute valuable insights for potential strategies to improve cancer treatment efficacy by utilizing these nanoparticles (Fig. 5).

## 4 Discussion

Colon cancer is a widespread form of cancer, and any new approach that could improve the effectiveness of treatment and reduce mortality rates is significant [21]. RT exerts its therapeutic effect by inducing DNA damage and subsequent cell death in cancer cells [22]. However, cancer cells can activate various DNA damage response mechanisms, leading to cell survival and radioresistance [23]. Therefore, augmenting the DNA damage induced by radiation can sensitize cancer cells to radiation therapy [24]. One approach to achieve this is through the use of nanoparticles, which can enhance the effect of radiation by increasing local radiation dose deposition and generating ROS [25].

In the last three decades, nanotechnology has revolutionized drug research, revealing new prospects in disease understanding and treatment [26,27].The utilization of metallic nanoparticles in medicine and disease diagnosis is on the rise, with gold nanoparticles being particularly popular due to their unique properties such as optical, electronic, and biocompatibility. These nanoparticles are able to penetrate cells more effectively due to their small size [28].

Nowadays, selenium nanoparticles have been used due to its important effects on health, especially the effects related to the immune response and cancer prevention activity [15].

Bi2Se3 NPs have been identified as a promising tool to enhance the efficacy of radiation therapy in treating colon cancer [29]. This discovery has been reported in several scientific articles, which have discussed the potential benefits of using Bi2Se3 NPs to increase the radiosensitivity of colon cancer cells.

The use of radiosensitizers has been a promising approach to enhance the effectiveness of radiotherapy and reduce the side effects associated with it [30]. Bi2Se3 NPs have shown potential as radiosensitizers [31,32], and this study investigated their potential in colon cancer cells.

The cell apoptotic rate was potentiated by the combination of Bi2S3@BSA-Met NPs with X-Ray irradiation [33]. Additionally, Bi@Bi2Se3 nanoparticles have been shown to enhance photothermal therapy for cancer cells by improving photothermal conversion and preventing oxidation [34]. CD47-targeted Bi@Bi2Se3 nanoparticles have also been found to improve photothermal therapy for cancer cells by increasing macrophage phagocytosis, demonstrating strong NIR absorbance, high conversion efficiency, good biocompatibility, and potential for in vivo tumor eradication [35]. With good biocompatibility and no significant toxicity in vitro and in vivo, Bi2Se3 nanoparticles have potential as a versatile theranostic for enhancing radiotherapeutic effects, reducing side-effects of radiation, and improving immune function [31].

The results showed that Bi2Se3 NPs significantly enhanced the sensitivity of colon cancer cells to ionizing radiation in a dose-dependent manner. This finding suggests that Bi2Se3 NPs have the potential to be used as a radiosensitizer in the treatment of colon cancer. The combination of Bi2Se3 NPs with radiation could enhance the effectiveness of radiotherapy and reduce the mortality rate associated with colon cancer. However, further studies are needed to investigate the safety and efficacy of this approach in vivo.

## 5 Conclusion

The combination of Bi2Se3 NPs and radiation therapy can be an advanced approach in the treatment of colon cancer and other malignancies. Notably, the low cellular toxicity of Bi2Se3 NPs in cultured cells renders them a reliable treatment adjunct. When combined with radiation therapy, these nanoparticles augment treatment efficacy by enhancing cellular radiation sensitivity, effectively functioning as radiation sensitizers. However, further comprehensive studies are essential to elucidate the intricate mechanism of action underlying their role as radiation sensitizers. It is imperative to highlight the necessity for clinical investigations to assess comprehensive safety and efficacy profiles of this compound, specifically regarding potential side effects on healthy cells.

In conclusion, employing Bi2Se3 NPs as radiosensitizers in colon cancer treatment shows promise for future investigation. The study's findings imply that integrating Bi2Se3 NPs with radiation could bolster radiotherapy effectiveness, potentially lowering colon cancer mortality rates. Subsequent in vivo research is necessary to ascertain the safety and efficacy of this approach. If proven safe and efficacious, it may substantially enhance the outcomes of radiotherapy for individuals with colon cancer.

## CRediT authorship contribution statement

**Hossein Khosravi:** Conceptualization. **Hamed Manoochehri:** Methodology, Formal analysis, Data curation. **Abbas Farmany:** Writing – original draft, Validation, Supervision. **Alireza Khoshghadam:** Visualization, Software, Investigation. **Hassan Rafieemehr:** Project administration, Methodology, Funding acquisition, Conceptualization. **Rasool Azmoonfar:** Writing – review & editing, Supervision.

## Declaration of competing interest

The researchers state that they have no conflict of interest.

## Data Availability

The data that has been used is confidential.
